# Parent-Child Physical Resemblance as Cues of Man’s Paternity

**DOI:** 10.5334/pb.424

**Published:** 2019-01-24

**Authors:** Barbara Dolinska

**Affiliations:** 1 Faculty of Social Sciences, Opole University, PL

**Keywords:** parent-child resemblance, Fatherhood, paternity, certainty, probability

## Abstract

The article presents the hypothesis that in the formation of judgements about a man’s biological fatherhood based on similarity of physical characteristics, people may take into consideration not only the similarity of father to child, but also of mother to child. The objective of the experiment was to conduct an initial investigation of that assumption. In the experiment, participants were presented with descriptions in which information was manipulated concerning the similarity of child to mother vs. to father vs. to neither of them. A total of 312 students of both sexes took participation in the experiment, having agreed to take part in a short psychological study immediately after classes were over. They were asked to read some short stories and to give their opinion as to whether the man described is the biological father of the child. It turned out that in conditions where the child’s appearance was dissimilar to both of the parents, the participants doubted the biological parenthood of the father. In conditions where the child was similar to the mother, the certainty of participants that the man was the biological father was as high as in conditions where the story indicated that the child was similar to him. The results thus suggest that information about a child’s similarity to its mother may, in some situations, be significant in the formulation of judgements on the biological fatherhood of a man.

## Introduction

In accordance with the evolutionary psychology paradigm, individuals face decisions on how to maximize their reproductive success (Clutton-Brock, & Vincent, 1991; Apicella, & Marlowe, 2004, 2007). In the case of all mammals, it is only the female that gives birth to offspring. This, of course, applies to humans as well, which means that women can be certain they are the biological parent of their children, whereas the situation of men is more complicated. Men either have no doubts, owing to trust in their partner (e.g. Buss & Schmitt, 1993; Geary, 2000), or are for various reasons not entirely certain as to their biological parenthood. In this case, they can follow various indications that the child is or is not a carrier of their genes. Psychologists in the evolutionary paradigm long ago assumed that in these cases men seek similarities to themselves in the child, and the more clearly they observe them, the greater material and non-material resources they invest in that child (Apicella, & Marlowe, 2004, 2007; Platek, Burch, Panyavin, Wasserman, & Gallup 2002; Platek, Critton, Burch, Frederick, Myers, & Gallup, 2003). Interestingly, this perceived similarity need not concern only external appearance, but also personality traits (Heijkoop, Dubas, & van Alken, 2009), interests, mannerisms and attitudes (Gallup, Ampel, Matteo, & O’Malley, 2016), and even body odour (Alvergne, Faurie, & Raymond, 2009). Other data also lend support to the theory that the possibility a man will physically abuse his wife and/or child is inversely proportional to the degree of similarity to him exhibited by that child (Burch & Gallup, 2000). Mothers seem to understand quite well that their partners’ convictions as to their own paternity are based on the child’s resemblance to themselves. Married women frequently begin to dream about their child’s being physically similar to their husbands already when they are pregnant (Leifer, 1977), and after birth they attempt to persuade their partner, that the child resembles its father more than its mother (Daly & Wilson, 1982). Studies also show that people who are not related to the man take into account the child’s resemblance to him when forming a judgement about his biological fatherhood (Oda, Matsumo-Oda, & Kurashima, 2002; McLain, Setters, Moulton, & Pratt, 2000). The reverse has also been observed: knowledge that a man is the father of a child influences perception of the similarity between those individuals (Bressan & Dal Martello, 2002; Oda, Matsumo-Oda, & Kurashima, 2005). It also turns out that the use of the cue concerning similarity when deciding about paternity is functional. In experiments by Bressan and Grassi (2004), participants viewed photos of a one-year-old child, estimated its similarity to three different people shown to them in photos, and then were asked to indicate which of those three people was the child’s parent (one person was, in fact, the child’s parent). When making this judgement, participants took into consideration which of the three people was physically most similar to the child, and the accuracy of their indications was much better than a random guess. We can thus say that the use of information about similarity of a man to a child in formulating judgements about biological fatherhood is of a universal nature: it applies to fathers, family members, and “external” observers.

The assumption that a man who is uncertain as to his fatherhood takes into consideration the degree of the child’s similarity to himself is, as we can see, well-documented empirically. In the present article, however, the hypothesis is advanced that the inferential processes that allow a man to draw such conclusions may be more complex. Indeed, it may be assumed that if a man who has any doubts as to his fatherhood declares that the child is not similar to him, he will seek other clues allowing him to resolve his doubts. This assumption will be elaborated upon in the present article by presenting an experiment that, owing to methodological considerations, will be capable of only indirectly verifying the presented arguments.

The preceding reasoning is based on the rather obvious assumption that people are aware that a child inherits physical features and psychological traits from both its father and its mother. In conditions in which the child is entirely dissimilar to the man, but is very similar to its mother, the man does not receive direct confirmation of the hypothesis that he is the biological father, but at the same time he also does not receive information contradicting that hypothesis. In other words, there is an absence of clues as to who the child’s biological father is. However, things are entirely different in the event the child resembles neither the presumed father, nor its mother. In this case, the man may not think that a specific physical features or psychological traits not inherited from him has instead been inherited from the mother. Just the opposite: he receives a signal that the feature or trait is neither his, nor the mother’s. If this is the case, then it is most certainly inherited from another man, who is the biological father of the child. The simplest example illustrating such a situation is that of a mixed-race couple. Let us assume that the man is white, and the mother is black. If the child is born white, the men receives information consistent with the hypothesis that he is the child’s biological father. If the child is born black, the man does not receive information entirely consistent with that hypothesis, but he also does not receive information negating it. He may thus accept the fact of his paternity, assuming that the child is simply physically similar to its mother. What, however, happens if the child has a yellow skin tone and facial features characteristic of Asians? This would obviously mean that the man receives information indicating that he is not the biological father of the child, who is entirely dissimilar to both him and its mother. In a very simple experiment it was demonstrated that people to whom such hypothetical situations were described in which the child is not similar to the father are convinced to a greater degree that the man is the father of the child if that child is similar (vs. dissimilar) to its mother (Dolinska, 2013b).

Although the above-mentioned study has generated results consistent with the assumptions presented above, it is worth noting that race is a very specific and unambiguous indicator which, by the same token, is an easy piece of information to employ in deducing one’s own paternity. The question arises, however, of whether the reasoning of the presumed father may take a similar course in conditions where more subtle features and cues must be taken into account, such as eye or hair colour. Can the conclusion that the child has eyes or hair of a different colour than its mother and her partner be for the former grounds to doubt that he is, in fact, the child’s biological father?

To answer directly the question of whether fathers wondering whether they are definitely raising their own child take into consideration not only similarity to themselves, but also to the mother, it would be necessary to study a group of men uncertain as to their fatherhood. In addition, a portion of them should be convinced that the child is similar to them, while another that the child is not similar to them but is to its mother, and yet another that the child is neither similar to them nor its mother. Such a study, apart from the incredible difficulty in identifying appropriate participants, would also involve very serious ethical complications. Merely asking men a question as to their fatherhood constitutes an encroachment into the privacy of the participant.

Unable therefore to directly check the adopted assumptions, decision was taken to use an indirect means of examining whether an element of similarity of a child to its mother can be used by a father in reasoning whether the child does or does not carry his genes. It was decided to present participants with one of several versions of a short story containing information about similarity of a child’s hair or eye colour to that of its mother and/or father. The participants’ task was to estimate the chances that the man presented in the story was the biological father of that child. In addition, participants were supposed to state what they thought the described man himself thought about the matter.^1^ It was decided that the experiment design should also take into account the sex of the child.

It was predicted that in conditions in which the child was not similar to the man, a decisive role in the judgement as to his biological fatherhood would be played by information about the child’s similarity to its mother. In this situation, information that the child is similar to its mother should lead to participants being more convinced that the man is the child’s biological father compared to when they learn that the child is not similar to the mother. The similarity mentioned above concerned either hair colour or eye colour. This distinction was introduced in order to exclude the occurrence of specific effects; it was assumed that the results patter should be the same in both cases. By the same token, it was not expected that information about the child’s sex would interact with the independent variables. No expectations were formulated in respect of the sex of participants.

## Study

### Participants

The participants were students of Wrocław University and Opole University, aged 18–46 (*M* = 21.58; *SD* = 2.74). A total of 312 people took part in the experiment (146 women and 166 men). Recruitment to the study was done by asking students directly after the conclusion of classes. Consent was given by approximately 80% of the students.

### Procedure

The experiment was designed in accordance with the ANOVA 2 (sex of participants) × 2 (sex of child in story) × 2 (physical features mentioned in the story: eye colour vs. hair colour) × 3 (similarity of child to parents: to father vs to mother vs to neither). Twelve versions of the story were thus created. Randomization was applied, allowing for a random assignment of versions of the story to study participants. Each participant read only one story. There were 26 people in each of the twelve experimental conditions (12 or 13 women and 13 or 14 men).^2^ The study was conducted in groups of 7 to 26 people. Participants were seated in a lecture hall at a distance from one another ensuring that they could not communicate nor learn that they had been given different stories. They were asked to spend two-three minutes participating in a study consisting in the completion of a short survey.

Participants were asked to read a short story about John, his partner Joanne, and the child (in half of the examples a girl, in the other half a boy) Joanne had given birth to. In half of the cases, the story described the eye colour of the threesome, while in the remaining it mentioned their hair colour. (Specific eye and hair colours were not used in the descriptions, as this would have required the experimental plan to be significantly expanded). Independently of this, distinct information was given as to the child’s resemblance to John vs. to Joanne vs. neither of the two. The exact wording of the story was thus:

*John K, a young man, has been for the last two years with a woman named Joanne. He is in love with her, and there is no visible evidence to suggest that Joanne is unfaithful to him. Joanne has given birth to a daughter/son. The girl/boy is healthy and of normal weight. John has eyes/hair of an entirely different colour than Joanne. The child has eyes/hair of the same colour as John/of the same colour as Joanne/of an entirely different colour than both John and Joanne*.

Participants were asked to judge the likelihood (in percent from 0% to 100%) that John was the biological father of the child, and then to respond to the question of to what extent John feels the same way.

Participants individually returned their completed surveys and left the lecture hall. A week later, at the next lecture, they were informed that the stories were false and the nature of the research was explained to them.

## Results

Initial analyses demonstrated that all continuous variables included in the model had a normal distribution, and it can be assumed that the variance in results of particular conditions are equal (Levene test for equality of variances: W = 1.993, p = .159 for the variable of likelihood that John is the child’s father, and W = 2.365, p = .125 for the expected judgement made by John of the likelihood that he was the biological father). The fulfilment of these assumptions led to the decision to analyse the results generated by the experiment using analysis of variance (ANOVA). A 2 (participant sex) × 2(child sex) × 2 (story version: “eyes” vs. “hair”) × 3 (child resemblance: to mother vs. to father vs. to neither of the parents) ANOVA on the dependent variable of likelihood that John is the child’s father yielded only two main effects: sex and resemblance. It turned out that women were more convinced than men that John was the biological father of child *F*(1,300) = 13.813; *p* < .001; *partial* η*^2^* = .044. The main effect of the factor of resemblance was also statistically significant: *F*(2,300) = 8.957; *p* < .001; *partial* η*^2^* = .056. It turned out that the participants were also almost equally certain that John was the biological father of the child in conditions where the child was not similar to him, but was similar to his partner; however, they were also clearly less certain when the child was not similar to either of those people (see Table 1). The remaining main effects and independent variable interactions were statistically insignificant (F < 1). A similar ANOVA on expected judgement made by John of the likelihood that he was the biological father revealed only one statistically significant effect. This was the main effect of the factor of resemblance: *F*(2,300) = 36,429; *p* < .001; *partial* η*^2^* = .195. This time the participants felt that John was most certain of his paternity in conditions where he perceived resemblance of the child to himself; less so when he does not see the child’s resemblance to himself but does know that the child is similar to his partner; and the lowest in conditions where the child was not similar to both him and his partner (see Table 1). The remaining main effects and independent variable interactions were statistically insignificant (F < 1).

**Table 1:** The average estimation of probability that John is the biological father of the child and judgments concerning John’s thinking about his own fatherhood.

	Participants’ opinion	Expected John’s opinion

Child resemblance to Father	M = 73.640*^a^*	M = 86.941*^b^*
	SD = 2.389	SD = 2.738
Child resemblance to mother	M = 71.380*^a^*	M = 77.378*^a^*
	SD = 2.370	SD = 2.716
Child resemblance to neither of the parents	M = 60.504*^c^*	M = 55.244*^d^*
	SD = 2.326	SD = 2.666

M = means; SD = standard deviations; Means which do not share a common superscript differ at .05 (Tukey’s HSD test).

The correlation between the personal conviction of the participants as to John’s paternity and their estimation of John’s certainty as to his fatherhood was statistically significant, but moderate (Pearson *r* = .342; *p* < .01). It also occurred that the participants felt John was more certain of his fatherhood (*M* = 72.917; *SD* = 30.415) than they themselves were of his paternity (*M* = 68,072; *SD* = 24.875); *t* (311) = 2.672; *p* < .008. This last dependency, however, was modified by information as to the resemblance of the child to John and his partner. In conditions in which the child was not similar either to John, nor to John’s partner, it was the participants’ opinion that he was less certain of his biological paternity (see Table 1)

## Discussion

The study confirmed what has been frequently noted in the psychological literature (e.g. Alexander, 1974; Burch & Gallup, 2000; Dolinska, 2013a; Platek et al, 2002) as to the association between physical resemblance of a man to a child born to his partner and certainty of that man that he is the child’s biological father. The results of the experiment presented above are consistent with this, while it should be emphasized that both technical and ethical considerations prevented fathers from being asked whether they were, in fact, the biological parents of children they were raising. As it turned out, people discussing the chances that someone else (the protagonist in the presented story) was the biological father of a child themselves take into account physical similarities between that person and the child. However, far more interesting is that we succeeded in our study in demonstrating that participants also took into consideration similarity of the child to its mother when drawing conclusions about the man’s fatherhood. If the child is not similar to either the father or the mother, then the participants are less certain that the man is the biological father than in conditions where the child is not similar to its father but is similar to its mother. However, it should be admitted that while the differences in these estimations were statistically significant, the effect size was rather small. It would seem that this results from the fact that participants were supplied with information about only one characteristic of external appearance (e.g., similarity or dissimilarity of hair or eye colour). It can be supposed that if similarity/dissimilarity were presented in a more global manner (e.g., as a set of features), both the differences between average estimations would be greater and (by the same token) the size effect would be clearly larger. Such clear distinctions were achieved by Dolinska (2013b) when presenting information on similarities and dissimilarities of skin colour among child, mother, and father.

Returning to the research presented in this article, it is worth emphasizing that the patterns of the obtained results were identical in conditions where the participants received information about hair colour and where the information concerned eye colour. While hair colour can undergo significant change (someone who is blond as a child can become a brunette in adolescence), apart from during the very first phase of life eye colour remains stable. The absence of differences in estimation of the certainty that the man is the child’s biological between the two aforementioned experimental conditions most likely results from the fact that the participants did not take the aforementioned facts into consideration.

It should also be pointed out that patterns of the results of participants were slightly different when responding to the question about their own opinion on the subject of John’s biological fatherhood than to the question of how John himself views the matter. Perhaps the participants concluded that distinguishing the two statements is appropriate because it attests to reflection in the course of performing the task and is expected by the researchers. To examine whether this assumption is correct, it would be necessary in future studies to introduce the attributive perspective (me vs character in the story) as an independent variable. A portion of the participants would then respond to a question about their own opinion, while the remaining would be asked about how the man in the story views the situation. If the suspicion presented here is correct, it should be expected that no differences will appear between those two experimental conditions.

It should also be emphasized that in real life situations people form judgements and suspicions about the biological paternity of a given father taking into account not only individual characteristics (as in the presented study), but an entire range of various physical features and psychological traits. They are also not limited to comparisons of the child with the mother and the (assumed) father, but they also look for similarities to grandparents and other family members. Awareness of the complexity involved in drawing conclusions about the biological paternity of a man does not, however, reduce the significance of the conclusion resulting from the research presented here: in conditions in which the child is not similar to a man, similarity of that child to its mother may play a role in the formation of judgements on the biological fatherhood of that man. It should be emphasized, however, that these judgements were formed not by men considering their own biological fatherhood, but by other people without even an emotional bond. In addition, the large majority of study participants (young students) were not parents, which may also have impacted the results obtained.

## Footnotes

The adoption of such a research method it also justified by other articles cited in the present text demonstrating that strangers also take into account a man’s similarity to a child when forming judgements about that man’s paternity (Oda, Matsumo-Oda, & Kurashima, 2002; McLain, Setters, Moulton, & Pratt, 2000).

Because neither effects of child sex nor of physical features were expected, N in each of the conditions 2 (sex of participants) × 3 (similarity of child to parents) was 48–56, which seems entirely sufficient to demonstrate differences between the averages, insofar as they in fact exist.
